# Prevalence, Distribution, and Risk Factor Correlates of High Thoracic Periaortic Fat in the Framingham Heart Study

**DOI:** 10.1161/JAHA.112.004200

**Published:** 2012-12-19

**Authors:** Kathryn A. Britton, Alison Pedley, Joseph M. Massaro, Erin M. Corsini, Joanne M. Murabito, Udo Hoffmann, Caroline S. Fox

**Affiliations:** 1 Division of Cardiovascular Medicine, Brigham and Women's Hospital, Boston, MA (K.A.B.); 2 Harvard Medical School, Boston, MA (K.A.B., U.H., C.S.F.); 3 National Heart, Lung and Blood Institute's Framingham Heart Study, Framingham, MA (K.A.B., A.P., J.M.M., C.S.F.); 4 Department of Mathematics and Statistics, Boston University, Boston, MA (J.M.M.); 5 Department of Radiology, Massachusetts General Hospital, Boston, MA (E.M.C., U.H.); 6 Department of Medicine, Section of General Internal Medicine, Boston University School of Medicine, Boston, MA (J.M.M.); 7 Division of Intramural Research and the Center for Population Studies, National Heart, Lung, and Blood Institute, Framingham, MA (C.S.F.); 8 Division of Endocrinology, Metabolism and Hypertension, Brigham and Women's Hospital and Harvard Medical School, Boston, MA (C.S.F.)

**Keywords:** body fat distribution, obesity, perivascular adipose tissue, risk factors, visceral adipose tissue

## Abstract

**Background:**

Thoracic periaortic adipose tissue (TAT) is associated with atherosclerosis and cardiovascular disease (CVD) risk factors and may play a role in obesity‐mediated vascular disease. We sought to determine the prevalence, distribution, and risk factor correlates of high TAT.

**Methods and Results:**

Participants from the Framingham Heart Study (n=3246, 48% women, mean age 51.1 years) underwent multidetector computed tomography; high TAT and visceral adipose tissue (VAT) were defined on the basis of sex‐specific 90th percentiles in a healthy referent sample. The prevalence of high TAT was 38.1% in women and 35.7% in men. Among individuals without high VAT, 10.1% had high TAT. After adjustment for age and VAT, both women and men with high TAT in the absence of high VAT were older and had a higher prevalence of CVD (*P*<0.0001) compared with those without high TAT. In addition, men in this group were more likely to be smokers (*P*=0.02), whereas women were more likely to have low high‐density lipoprotein cholesterol (*P*=0.005).

**Conclusions:**

Individuals in our community‐based sample with high TAT in the absence of high VAT were characterized by an adverse cardiometabolic profile. This adipose tissue phenotype may identify a subset of individuals with distinct metabolic characteristics.

## Introduction

Obesity is associated with cardiovascular morbidity and mortality.^1^ The mechanisms by which obesity might contribute to vascular disease remain incompletely understood. Body fat distribution may be a cardiovascular risk factor even after accounting for generalized adiposity.^2^ One component of abnormal body fat deposition involves the deposition of adipose tissue, so‐called ectopic fat, around organs and the vasculature.^3^ Perivascular fat is one such ectopic fat depot that has been postulated to have a local pathogenic effect on blood vessels.^4–8^ Thoracic periaortic fat is a subtype of perivascular fat that can be quantified using multidetector computed tomography (MDCT).^9^

Thoracic periaortic fat (TAT) may be a novel risk marker for cardiovascular disease.^10–11^ We have previously shown that TAT is associated with certain metabolic risk factors after adjustment for body mass index (BMI) as well as abdominal aortic and coronary calcium after adjustment for either BMI or visceral adipose tissue (VAT).^11^ However, the prevalence and age distribution of TAT in a community‐based sample has not been described. In addition, given the known correlation between TAT and VAT^11^ and the known association of VAT with cardiometabolic risk,^12^ we sought to examine the relative association of high TAT versus high VAT with cardiometabolic risk by examining different patterns of thoracic periaortic and visceral fat deposition in a cohort of middle‐aged individuals enrolled in the Framingham Heart Study.

## Methods

In 1971, children of those in the original Framingham Heart Study cohort and their spouses were enrolled in the Offspring cohort. In 2002, individuals with at least 1 parent in the Offspring cohort were enrolled in the Third‐Generation cohort. The study designs have been described previously.^13–14^ The present study included participants from the Offspring and Third‐Generation cohorts who participated in the MDCT substudy between 2002 and 2005 as previously described.^12^ Of the 3529 participants in the MDCT substudy, 3246 had interpretable values for TAT. Of the individuals with interpretable TAT, 3228 had interpretable values for VAT. For individual regressions, any individuals with data missing the covariate of interest were excluded. The study protocol was approved by the institutional review boards of the Boston University Medical Center and Massachusetts General Hospital. All subjects provided written informed consent.

### MDCT Scan Protocol and Adipose Tissue Measurements

Participants underwent radiographic assessment of their thorax and abdomen in the supine position using an 8‐slice MDCT scanner (LightSpeed Ultra, General Electric, Milwaukee, WI) as previously described.^9^ The thoracic scan was performed during an inspiratory breath hold with prospective ECG triggering (with the center of acquisition window at 70% of the R‐R cycle to minimize cardiac motion). The average scan time was 18 seconds (tube voltage of 120 kVp, tube current of 320 mA (<220 lbs) or 400 mA (>220 lbs) with a gantry rotation time of 500 ms and a temporal resolution of 330 ms. Thoracic and abdominal MDCT images were reconstructed as 2.5‐ and 5‐mm nonoverlapping slices, respectively.

**Figure 1.**
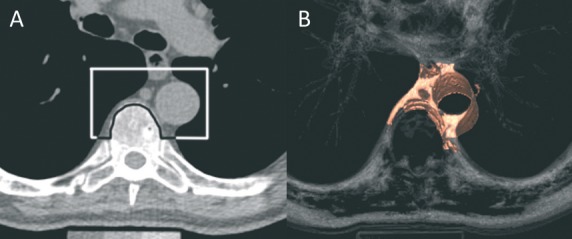
A, The region of interest drawn around the aorta using anatomic landmarks. Adipose tissue within this region of interest, defined as pixels with Hounsfield units between −195 and −45, is considered periaortic fat. B, 3D reconstruction. Reproduced from Fox et al (10) with permission from the publisher.^10^

TAT and VAT were assessed using a dedicated workstation (Aquarius 3D, TeraRecon, San Mateo, CA). Fat volumes were measured by a semiautomatic segmentation technique requiring manual definition of tissue borders. Fat within an area of interest was defined by pixels with characteristic Hounsfield units (HU; window width −195 to −45 HU; window center −120 HU). The area of interest for TAT was defined anteriorly by the area immediately surrounding the thoracic aorta (defined by a line drawn horizontally through the esophagus, which connected to the left costovertebral joint) and posteriorly by the right lateral border of the vertebral body and the anterior edge of the vertebral body.^9^ This resulted in a 6.75‐cm column of fat (27 slices) surrounding the thoracic aorta. VAT was quantified on abdominal scans as previously described.^12^ Briefly, the reader defined the area of interest by tracing the abdominal muscular wall and separating the subcutaneous from the visceral abdominal fat depot. These areas of interest were summed over the 25 abdominal slices. Intrareader and interreader (assessed between 2 readers) reproducibility was excellent for both TAT and VAT, with intraclass and interclass correlations >0.98.^9,15^ A visual representation of the methodology for measuring TAT is presented in Figure 1.

### Covariate Assessment

Covariates were measured at the seventh Framingham Offspring examination (1998–2001) and the first Third‐Generation examination (2002–2005). BMI was defined as weight (in kilograms) divided by the square of the height (in meters). Waist circumference (WC) was measured at the level of the umbilicus. Current smoking was defined as smoking ≥1 cigarette per day in the past year. Alcohol use was assessed by physician‐administered questionnaires, and dichotomized on the basis of consumption of ≥14 drinks per week (in men) or ≥7 drinks per week (in women). A physical activity index (PAI) score was calculated by summing the reported numbers for each level of activity, weighted by their estimated metabolic expenditure, as described previously.^16^ The PAI ranges from a minimum score of 24, indicating 24 hours of sleeping, to a maximum score of 120, indicating 24 hours of heavy physical activity. Serum triglycerides, total and high‐density lipoprotein cholesterol, and fasting plasma glucose were measured on fasting morning samples. Fasting plasma glucose ≥126 mg/dL or treatment with a hypoglycemic agent or insulin was used to define diabetes mellitus. Hypertension was defined as a systolic blood pressure ≥140 mm Hg, diastolic blood pressure ≥90 mm Hg or treatment with an antihypertensive agent. Metabolic syndrome was defined from the modified Adult Treatment Panel criteria.^17^ If periods had stopped for >1 year, women were considered postmenopausal. Cardiovascular disease included coronary heart disease, stroke, intermittent claudication, and congestive heart failure.

### Statistical Analysis

To determine the prevalence of high TAT, a healthy referent sample was created by hierarchical exclusion of participants with the following covariates: BMI≥30 kg/m^2^ (n=874), presence of hypertension (or use of antihypertensive medications; n=562), triglycerides ≥150 mg/dL or lipid‐lowering medication (n=424), low high‐density lipoprotein (HDL) cholesterol (<40 mg/dL in men and <50 mg/dL in women; n=193), impaired fasting glucose, diabetes or use of hypoglycemic medications (n=215); prevalent cardiovascular disease (CVD; n=9); current tobacco smoking (n=118); BMI<18.5 kg/m^2^ (n=12), and missing covariates (n=24), resulting in 499 women and 316 men. We defined high TAT as a sex‐specific fat volume ≥90th percentile from the healthy referent sample. Sex‐specific TAT volumes <90th percentile from the healthy referent sample were classified as normal. The 90th percentile was chosen to ensure adequate sample size in the healthy referent sample to provide statistically robust estimates for the fat volume cutoff values. This method used to determine the volume for high TAT was the same as that used for other fat depots in the Framingham Heart Study.^18–19^ High TAT, by this definition, corresponded with volumes ≥10.2 cm^3^ in women and ≥19.0 cm^3^ in men. Prevalence estimates of high TAT were then determined in the overall sample (including those individuals who had been excluded from the healthy referent sample) and stratified by sex and the following age categories: 35 to 44, 45 to 54, 55 to 64, 65 to 74, and 75 to 84 years.

To establish the risk factor profiles associated with different patterns of TAT and VAT, we stratified our sample into 4 mutually exclusive categories on the basis of TAT and VAT volumes: (A) normal TAT and normal VAT, (B) high TAT but normal VAT, (C) high VAT and normal TAT, and (D) high TAT and high VAT. High VAT has been previously defined as ≥1359 cm^3^ in women and ≥2323 cm^3^ in men. Given the known correlation between TAT and VAT,^10^ we examined differences in risk factor levels between the discordant categories (groups B versus C) using sex‐specific age‐adjusted analyses of covariance (ANCOVAs) and logistic regressions. Given that high VAT is already recognized as associated with an adverse cardiometabolic risk factor profile, we also examined differences in risk factors among individuals with normal VAT that were discordant for high TAT (groups A versus B). For these analyses, we used sex‐specific ANCOVAs and logistic regressions adjusted for age as well as the volume of VAT. We also adjusted for VAT given that individuals with high TAT and normal VAT tended to have higher absolute volumes of VAT compared with those with normal TAT and normal VAT. We did not adjust for height given the lack of documented association between height and TAT (*r*=0.05, *P*=0.25 in women; *r*=0.01, *P*=0.75 in men)^11^ We tested for an age interaction across the 4 different categories of TAT/VAT. When age interactions were significant, we performed additional analyses stratified by median age. Finally, we performed a sensitivity analysis restricting our sample to the Third‐Generation cohort in whom risk factor assessment and MDCT scans were performed during the same period.

SAS version 9.1 was used to perform all computations. Two‐sided *P* values <0.05 were considered significant. Because of the exploratory nature of this study, no adjustments were made for multiple comparisons.

## Results

Overall, 1558 women and 1688 men for a total sample of 3246 individuals were included in this analysis. For the overall sample, the mean values for TAT in women and men were 9.9 and 17.5 cm^3^, respectively. The characteristics of the sample population are described in Table 1.

**Table 1.** Clinical Characteristics of the Overall Sample*

Characteristic	Overall (N=3246)	Women (n=1558)	Men (n=1688)
Age, y	51.1±10.4	52.3±9.9	49.9±10.7
BMI (kg/m^2^)	27.7±5.2	27.0±5.8	28.4±4.5
Waist circumference (cm)	97.1±14.2	93.0±15.5	100.8±11.7
Low HDL cholesterol* (%)	29	26	33
Elevated triglycerides* (%)	36	27	44
Smoking status (%)			
Current	13	12	13
Former	39	43	37
Never	48	45	50
Alcohol use* (%)	15	15	16
Physical activity index	37.6±7.2	36.7±5.8	38.3±8.2
Postmenopausal (%)	—	51	—
Hypertension (%)	29	27	32
Impaired fasting glucose* (%)	29	19	38
Diabetes (%)	7	6	7
Metabolic syndrome (%)	33	27	38
CVD (%)	6	4	8
Hypertension treatment (%)	19	19	20
Lipid treatment (%)	14	10	18
TAT (cm^3^)	13.8±8.2	9.9±5.3	17.5±8.7
VAT (cm^3^)	1818.7±1034.2	1356.7±825.3	2243.2±1025.0

Data are presented as mean±SD for continuous or % for categorical characteristics.

BMI indicates body mass index; HDL, high‐density lipoprotein; CVD, cardiovascular disease; TAT, thoracic periaortic fat; VAT, visceral adipose tissue.

* Sample sizes vary from row to row, as available data were used for a given characteristic.

* Defined as <40 mg/dL (men) and <50 mg/dL (women).

* Defined as ≥150 mg/dL or lipid treatment.

* Defined as ≥14 drinks weekly for men and ≥7 drinks weekly for women.

* Defined as fasting plasma glucose 100 to 125 mg/dL and not currently taking diabetes medication.

### Distribution of Thoracic Periaortic Fat in the Community

Sex‐ and age‐specific TAT volumes are represented in Table 2. TAT volumes increased with age into the eighth decade for women and into the seventh decade for men for all percentiles of fat.

**Table 2.** Thoracic Periaortic Fat Percentiles Within Age Groups Among Women (n=1543) and Men (n=1634)*

	Fat Volumes, cm^3^							
	N	5th	10th	25th	50th	75th	90th	>95th
Women (age), y								
35 to 44	391	3.2	3.7	4.8	6.3	8.8	11.4	13.8
45 to 54	591	3.9	4.4	5.8	8.1	11.3	15.4	17.7
55 to 64	341	5.2	6.0	7.9	10.8	14.1	17.7	20.6
65 to 74	185	6.4	7.1	9.7	13.1	17.7	22.6	25.1
75 to 84	35	7	7.6	11.0	14.5	19.3	26.5	28.2
Men (age), y								
35 to 44	559	5.9	6.7	9.5	12.5	16.9	22.0	26.8
45 to 54	582	7.2	8.9	11.7	15.2	20.1	25.2	29.5
55 to 64	280	9.8	11.7	15.1	20.2	26.3	31.8	38.7
65 to 74	182	11.1	12.6	17.5	23.6	31.7	39.6	43.9
75 to 84	31	7.9	13.2	16.2	23.1	34.5	42.7	43.9

* Fifteen women and 54 men from the total sample were either <35 years of age or missing information on age and therefore were excluded from this analysis.

### Prevalence of High Thoracic Periaortic Fat

The overall and age‐stratified prevalence of high TAT, defined as ≥90th percentile in the healthy referent sample, is reported in Table 3. The overall prevalence for high TAT was 38.1% in women and 35.7% in men and increased with age (*P* value for linear trend <0.0001). As expected, the prevalence of high TAT increased with increasing BMI and waist circumference categories (Table 4).

**Table 3.** Sex‐Specific Prevalence (Standard Error) of Excess TAT* by Age Group

	Women (n=1543)*	Men (n=1634)*
Overall	38.1% (1.24)	35.7% (1.19)
35 to 44	15.3% (1.82)	17.9% (1.62)
45 to 54	32.7% (1.93)	29.4% (1.89)
55 to 64	52.8% (2.70)	57.5% (2.95)
65 to 74	69.2% (3.39)	72.0% (3.33)
75 to 84	77.1% (7.10)	67.7% (8.40)
*P* value for linear trend	<0.0001	<0.0001

TAT indicates thoracic periaortic fat.

* Excess TAT defined as ≥90 percentile of the sex‐specific cut points (≥10.2 and ≥19.0 cm^3^ in women and men, respectively) in a healthy referent sample.

* Fifteen women and 54 men from the total sample were either <35 years of age or missing information on age and therefore were excluded from this analysis.

**Table 4.** Sex‐Specific Prevalence (Standard Error) of High TAT* by BMI and Waist Circumference Category

BMI Category	Women (n=1544)	Men (n=1674)	Waist Circumference Category	Women (n=1539)	Men (n=1671)
Normal weight (BMI<25 kg/m^2^)	12.4% (1.26)	6.6% (1.30)	Normal waist circumference (≤88 cm in women, ≤102 cm in men)	9.8% (1.16)	16.2% (1.18)
Overweight (25 kg/m^2^≤BMI<30 kg/m^2^)	43.7% (2.28)	29.7% (1.59)	High waist circumference (>88 cm in women, >102 cm in men)	58.5% (1.66)	60.8% (1.85)
Obese (BMI≥30 kg/m^2^)	75.4% (2.18)	65.0% (2.17)			

TAT indicates thoracic periaortic fat; BMI, body mass index.

* High TAT defined as ≥90 percentile sex‐specific cut points (≥10.2 and ≥19.0 cm^3^ in women and men, respectively) for TAT in a healthy referent sample.

### Cardiometabolic Risk Factor Profiles by Patterns of Thoracic Periaortic Fat and Visceral Adipose Tissue

Given the known high correlation between TAT and VAT (*r*=0.75, *P*<0.001),^11^ we divided individuals into 4 categories on the basis of the presence or absence of high TAT and the presence or absence of high VAT to better evaluate the unique correlates of each fat depot. We then compared cardiometabolic risk factors between individuals with high TAT and normal VAT versus high VAT and normal TAT. In addition, among individuals with normal VAT, we compared cardiometabolic risk factors among individuals discordant for high TAT.

In the overall sample, 16.4% of women and 18.8% of men were discordant for high TAT and high VAT. Comparing discordant groups, women with high TAT and normal VAT compared with high VAT and normal TAT were older but tended to have lower measures of clinical adiposity and better cardiometabolic risk factor profiles after adjusting for age (Table 5). In contrast, men with high TAT and normal VAT compared with high VAT and normal TAT were older and had a lower waist circumference, but otherwise the cardiometabolic profiles were not significantly different (Table 5).

**Table 5.** Age‐Adjusted Sex‐Specific Distribution of Risk Factors and Clinical Characteristics by VAT/TAT Categories* Among Women (n=1546) and Men (n=1682)*

Risk Factor	Normal VAT/Normal TAT (A)	High VAT/Normal TAT (B)	High TAT/Normal VAT (C)	High VAT/High TAT (D)	Age‐Adjusted *P* Value Comparing Groups B and C
Women					
n	787	168	86	505	
Age (y)	48.7	51.6	57.3	57.2	<0.0001
BMI (kg/m^2^)	23.4	29.2	26.2	32.1	<0.0001
Waist circumference (cm)	83.4	99.6	89.8	106.6	<0.0001
VAT (cm^3^)	750.5	1693.6	1082	2236.4	<0.0001
TAT (cm^3^)	6.5	8.6	12.1	15.1	<0.0001
Diabetes (%)	2.6	5.8	4.0	8.8	0.50
Impaired fasting glucose* (%)	8.6	24.2	12.8	32.7	0.03
Hypertension (%)	16.6	31.7	21.1	39.5	0.049
Elevated triglycerides* (%)	12.8	34.9	28.3	44.1	0.27
Low HDL cholesterol* (%)	12.3	35.0	27.7	44.7	0.25
Metabolic syndrome (%)	5.9	38.0	17.4	55.6	0.0006
CVD (%)	1.5	4.0	7.5	6.2	0.20
Smoking status (%)					
Current	9.9	12.3	17.4	16.7	0.29
Former	42.2	37.0	41.9	45.4	0.44
Never	48.2	50.8	41.1	38.7	0.15
Men					
n	876	216	101	489	
Age (y)	46	49.4	55.4	56.0	<0.0001
BMI (kg/m^2^)	25.9	29.6	29.0	32.3	0.17
Waist circumference (cm)	94.2	104.7	102.0	110.6	0.017
VAT (cm^3^)	1525.4	2788.6	1994.5	3340.6	<0.0001
TAT (cm^3^)	12.2	15.4	22.4	26.6	<0.0001
Diabetes (%)	3.7	9.0	7.0	11.4	0.53
Impaired fasting glucose* (%)	29.7	40.3	39.8	49.5	0.92
Hypertension, %	22.5	33.0	35.7	45.4	0.62
Elevated triglycerides* (%)	31.1	57.3	56.2	57.7	0.85
Low HDL cholesterol* (%)	22.5	41.7	40.2	46.2	0.80
Metabolic syndrome (%)	17.0	51.8	45.7	66.9	0.32
CVD (%)	4.6	8.5	11.7	9.6	0.32
Smoking status (%)					
Current	11.3	14.0	18.6	16.3	0.31
Former	33.1	35.5	38.8	42.1	0.55
Never	55.5	50.9	43.0	41.4	0.19

VAT indicates visceral adipose tissue; TAT, thoracic periaortic fat; BMI, body mass index; HDL, high‐density lipoprotein; CVD, cardiovascular disease.

* TAT and VAT categories are defined as high if ≥90th percentile sex‐specific cut points in healthy referent sample. High TAT corresponded to volumes ≥10.2 and ≥19.0 cm^3^ in women and men, respectively. High VAT has been previously defined as ≥1359 cm^3^ in women and ≥2323 cm^3^ in men.

* Sample sizes vary from row to row, as available data were used for a given characteristic.

* Defined as fasting plasma glucose 100 to 125 mg/dL and not currently taking diabetes medication.

* Defined as ≥150 mg/dL or lipid treatment.

* Defined as <40 mg/dL (men) and <50 mg/dL (women).

**Figure 2.**
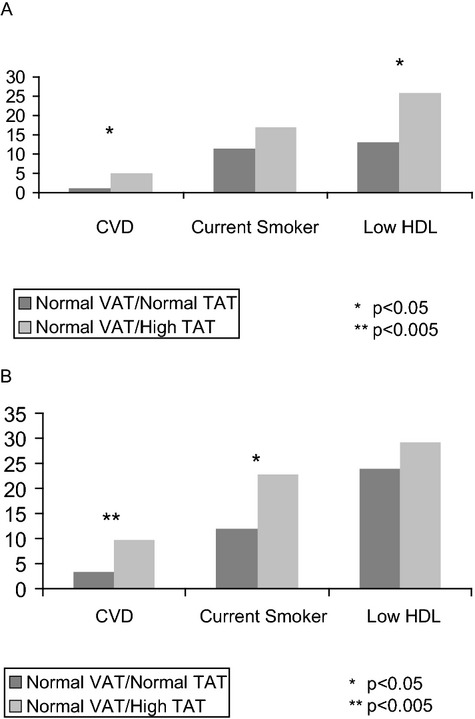
Differences in cardiometabolic features among (A) women (n=873) and (B) men (n=977) with normal VAT/normal TAT vs normal VAT/high TAT. Prevalence rates and *P* values are adjusted for age and volume of VAT. CVD indicates cardiovascular disease; HDL, high‐density lipoprotein; TAT, thoracic periaortic adipose tissue; VAT, visceral adipose tissue.

Among individuals with normal VAT, high TAT compared with normal TAT was associated with a more adverse cardiometabolic profile. Specifically, these individuals were older and had a higher prevalence of the majority of cardiometabolic risk factors, including a higher prevalence of metabolic syndrome (Table 5). Findings were similar when we also adjusted for BMI (data not shown). However, individuals with high TAT and normal VAT also had a higher absolute volume of VAT compared with individuals with normal TAT and normal VAT. Given this difference in VAT volumes, our findings presented in Figure 2 reflect additional adjustment of these models for the absolute volume of VAT. In these models, the presence of high TAT was associated with prevalent CVD in both sexes (age‐ and VAT‐adjusted *P*=0.01 in women, Figure 2A; and *P*=0.004 in men, Figure 2B). In addition, among women with normal VAT, high TAT was associated with significantly lower HDL levels (*P*=0.005) (Figure 2A). In men with high TAT and normal VAT, there was a higher prevalence of smoking (age‐VAT‐adjusted *P*=0.02) and higher BMI (*P*=0.004) compared with individuals with normal VAT and normal TAT. After adjustment for the volume of VAT, the difference in the prevalence of metabolic syndrome was no longer different between those with high TAT versus normal TAT. When we reexamined the association of high TAT versus normal TAT with CVD prevalence after adjustment for low HDL and current smoking (in addition to adjustment for age and volume of VAT), there remained a significant association in both women (*P*=0.02) and men (*P*=0.01).

There were multiple significant age interactions across the 4 categories of TAT/VAT categories. Thus, we performed analysis stratified by median age. The results were generally similar to the overall findings (data not shown). Finally, in our sensitivity analysis limited to the Third Generation cohort, who underwent risk factor and MDCT assessment during the same time period, findings were overall similar (Table 6).

**Table 6.** Age‐Adjusted Sex‐Specific Distribution of Risk Factors and Clinical Characteristics by VAT/TAT Categories* Among Women (n=848) and Men (n=1038) Limited to the Third Generation*

Risk Factor	Normal VAT/Normal TAT (A)	High VAT/Normal TAT (B)	High TAT/Normal VAT (C)	High VAT/High TAT (D)	Age‐Adjusted *P* Value Comparing Groups B and C
Women					
n	518	54	66	210	
Age (y)	45.3	46.2	47	48.2	0.40
BMI (kg/m^2^)	23.4	30.2	26.3	33.2	<.0001
Waist circumference (cm)	82.3	99.8	90.9	108.2	<.0001
VAT (cm^3^)	688.8	1653.8	1037.9	2125	<.0001
TAT (cm^3^)	5.7	7.5	10.2	13.1	<.0001
Diabetes (%)	1.5	5.6	1.4	6.1	0.21
Impaired fasting glucose* (%)	5.9	24.1	10.2	30.4	0.04
Hypertension (%)	10.0	25.9	15.2	31.1	0.13
Elevated triglycerides* (%)	6.6	22.2	25.1	39.1	0.71
Low HDL cholesterol* (%)	14.5	38.9	24.7	47.3	0.10
Metabolic syndrome (%)	2.8	28.2	14.5	52.3	0.06
CVD (%)	0.2	1.9	0.0	3.0	0.95
Smoking status					
Current	11.7	16.7	15.8	19.9	0.90
Former	36.6	27.8	41.6	36.2	0.12
Never	51.9	55.6	42.5	44.4	0.16
Men					
N	585	85	130	238	
Age (y)	42.5	44.4	45.5	46.4	0.22
BMI (kg/m^2^)	25.9	30	28.1	32.7	0.0001
Waist circumference (cm)	93.3	104.7	100.4	111.5	0.0007
VAT (cm^3^)	1424.7	2744.4	1940.7	3094.9	<.0001
TAT (cm^3^)	10.5	13.5	18.7	23.2	<.0001
Diabetes (%)	2.5	9.3	1.3	8.1	0.01
Impaired fasting glucose* (%)	25.6	40.2	41.4	46.5	0.86
Hypertension (%)	15.1	35.8	26.7	40.4	0.145
Elevated triglycerides* (%)	26.9	62.1	53.1	58.1	0.19
Low HDL Cholesterol* (%)	20.2	54.5	35.7	42.5	0.01
Metabolic syndrome (%)	12.9	59.7	37.5	67.3	0.002
CVD (%)	2.2	4.6	0.7	3.1	0.08
Smoking status (%)					
Current	12.8	8.3	18.8	21.2	0.04
Former	23.9	27.3	27.3	28.6	0.99
Never	63.3	64.3	54.1	50.2	0.13

VAT indicates visceral adipose tissue; TAT, thoracic periaortic fat; BMI indicates body mass index; CVD, cardiovascular disease; HDL, high‐density lipoprotein.

* TAT and VAT categories are defined as high if ≥90th percentile sex‐specific cut points in healthy referent sample. In the Third Generation, high TAT corresponded to volumes ≥8.79 and 15.72 cm^3^ in women and men, respectively. High VAT has been previously defined as ≥1359 cm^3^ in women and ≥2323 cm^3^ in men.

* Sample sizes vary from row to row, as available data were used for a given characteristic.

* Defined as fasting plasma glucose 100 to 125 mg/dL and not currently taking diabetes medication.

* Defined as ≥150 mg/dL or lipid treatment.

* Defined as <40 mg/dL (men) and <50 mg/dL (women).

## Discussion

In our community‐based sample, more than a third of individuals had high TAT. In the absence of high VAT, excess TAT identified a subset of individuals with a higher prevalence of adverse cardiometabolic characteristics compared with individuals without high TAT even after adjustment for total volume of VAT. This included a higher prevalence of CVD in both women and men, lower HDL levels in women, and a higher prevalence of current smoking in men. These findings provide a better understanding of the risk factor correlates of TAT, a specific subtype of perivascular fat. Further experimental studies are required to elucidate whether TAT is pathogenic.

Multiple basic science and small clinical studies have suggested a local effect of perivascular fat that changes with the development of obesity.^5–6,8^ Previous work in the Framingham Heart Study has demonstrated a novel and reliable method of quantifying thoracic periaortic fat by MDCT.^9^ We now present a comprehensive age‐ and sex‐specific description of TAT distribution in a community‐based sample and report differences in cardiometabolic features among subcategories of TAT and VAT.

Our findings that more than one third of individuals in the community had high TAT is similar to our previous reports of fat depots, including VAT, pericardial, and intrathoracic fat.^18–19^ Despite this similar prevalence among various fat depots, our findings suggest that a high volume of TAT is associated with adverse cardiometabolic features among the subset of individuals with normal VAT. Prior work has highlighted the existence of “metabolically obese normal weight individuals” who exhibit glucose intolerance and hyperinsulinemia despite a normal BMI.^20^ Differences in visceral adiposity have been postulated to contribute to this phenotype.^21^ Our findings suggest that high thoracic periaortic fat may also identify a “metabolically obese” group among individuals who do not meet criteria for excess VAT. Some of these differences, such as the higher prevalence of CVD, are present in both sexes. However, others are sex specific, with a prevalence of low HDL higher in women than men. This finding is consistent with prior literature suggesting stronger associations of ectopic fat and metabolic risk factors in women compared with men. However, the prevalence of current smoking was higher in men than in women with high TAT/normal VAT compared with normal TAT/normal VAT.

TAT is directly wrapped around the aorta, and this distinct anatomic location may explain the specific association between high TAT and CVD among individuals with normal VAT. TAT may serve as a marker of perivascular fat throughout the body including smaller blood vessels, and perivascular fat has been postulated to have adverse effects on the vasculature.^4–5^ Supporting this, prior work in the Framingham Heart Study has demonstrated an association between TAT and both abdominal aortic calcium and coronary artery calcium among individuals without known cardiovascular disease. These associations persisted after adjustment for VAT and standard cardiovascular risk factors.^10^ Alternatively, our findings that high TAT was associated with CVD among individuals with normal VAT may reflect that these individuals were more likely to have a higher prevalence of certain CVD risk factors, including low HDL in women and smoking in men.

Our findings of a difference in the prevalence of smoking among men with normal VAT but discordant for TAT deserves specific comment. Smokers tend to have a lower body weight but are known to have a higher risk of cardiovascular disease. Ectopic fat distribution, specifically higher volumes of VAT, is already known to differ between smokers and nonsmokers.^22^ We have extended these findings among individuals with normal VAT by demonstrating a higher prevalence of male smokers among individuals with high versus normal TAT. These findings are noteworthy given the potential modulating role of nicotine on perivascular fat previously demonstrated in animal models.^23^ Specifically, exposure of rats to nicotine prenatally and during lactation led to an increase in total adiposity as well as perivascular fat in the offspring compared with controls. Furthermore, the normal anticontractile effect of perivascular fat on blood vessels was no longer present in nicotine‐exposed offspring, but was restored with the transfer of the medium surrounding normal fat. Thus, nicotine was associated with higher volumes as well as dysfunction of perivascular fat. These findings are consistent with prior work suggesting that excess perivascular adipose tissue disrupts the normal contribution of perivascular fat to vascular tone^24^ and further suggest that nicotine may contribute to this process. Other studies also support a potential role of nicotine on adipose tissue.^25–26^ For example, differences in adipose tissue lipoprotein lipase activity have been found between smokers and nonsmokers.^25^ Thus, elucidation of the full expression profile of perivascular fat in response to nicotine may help to extend our observational findings.

### Strengths and Limitations

The major strength of our study is the relatively large sample size and community‐based nature of the cohort. This allowed exploration of differences within subgroups of TAT and VAT and limited referral bias. We assessed TAT and VAT using a highly reproducible CT volumetric assessment. Certain limitations warrant discussion. The cross‐sectional and observational design of the analysis prevents inferences of causality or temporality. The Framingham Heart Study is predominantly white, and results cannot be generalized to other ethnic groups. We did not have information on the severity of CVD. Our analyses were exploratory, and we did not account for multiple testing. Replication of our findings in independent cohorts is warranted. There were temporal differences between the MDCT scans and the risk factor assessments among the Offspring cohort. However, our sensitivity analysis limited to the Third‐Generation cohort (in which MDCT scans and risk factor assessment occurred during the same period) demonstrated overall similar findings. Finally, our findings do not suggest that TAT quantification should be used as a clinical tool.

## Conclusions

Elevated TAT is prevalent in the community and is associated with adverse cardiometabolic features, including CVD, smoking, and low HDL among individuals with normal VAT. Further work to better understand the biology of TAT and its association with metabolic and cardiovascular disease may provide insight into a potentially unique pathogenic role of periaortic fat.
